# Erfolgreiche Reduzierung der NAPPA-Scores (Nail Assessment in Psoriasis and Psoriasis-Arthritis) mit Secukinumab bei schwerer Nagelpsoriasis

**DOI:** 10.1007/s00105-023-05235-1

**Published:** 2023-10-12

**Authors:** Aminah Alhumam

**Affiliations:** 1 Institut für Versorgungsforschung in der Dermatologie und bei Pflegeberufen (IVDP), Universitätsklinikum Hamburg-Eppendorf (UKE), Martinistr. 52, 20246 Hamburg, Deutschland; 2 Department of Dermatology, College of Medicine King Faisal University, Al-Ahsa, Saudi-Arabien

## Anamnese

Wir berichten über einen 38-jährigen Patienten mit einem ausgeprägten Befund von einer Psoriasis vulgaris, asymptomatischer Psoriasisarthritis und deutlich refraktärer Nagelpsoriasis der Finger und Zehen, die als primäre Erkrankung bereits in der Kindheit auftrat. Die asymptomatische Psoriasisarthritis wurde vor einigen Jahren diagnostiziert. Er stellte sich nach mehreren erfolglosen topischen und systemischen Therapien vor. Als Begleiterkrankung ist eine arterielle Hypertonie bekannt, die mit Candesartan behandelt wird.

Vor Beginn der Secukinumab-Behandlung hatte der Patient viele verschiedene Therapien erhalten, z. B.: Bade-PUVA (Psoralen und ultraviolette Strahlung [UV-A]; kurzzeitig, unzureichender Therapieerfolg), Ciclosporin A (< 10 Monate, Nebenwirkung: Kreatininanstieg), Fumarsäureester (< 12 Monate, wegen Lymphopenie abgesetzt), Methotrexat (> 12 Monate, Verschlechterung des Hautbefundes bei sehr gutem Ansprechen auf Psoriasisarthritis) und Adalimumab (> 6 Monate; unzureichendes Ansprechen auf den Hautbefund).

## Klinischer Befund

Bei der Untersuchung fanden wir münzgroße bis landkartenartige konfluierende erythematosquamöse Plaques mit ausgeprägtem Erythem, mäßiger Infiltration und grob lamellarer, weißlicher, anhaftender Schuppung am ganzen Körper. Der Patient wies einen Psoriasis Area and Severity Index (PASI) von 31,3/72 und einen Dermatology Life Quality Index (DLQI) von 17/30 auf.

Die Nägel zeigten ausgeprägte psoriasiforme Nagelveränderungen in Form von Leukonychien, Onycholyse, Lochfraß, Öltropfen und subungualer Hyperkeratose an allen Finger- und Zehennägeln. Der NAPPA-Score (Nail Assessment in Psoriasis and Psoriatic Arthritis) zeigte vor der Therapie: NAPPA-QOL (Quality of Life) 55/80, NAPPA-PBI (Patient Benefit Index) 3/96, NAPPA-CLIN (Clinic) 16/16.

## Therapie und Verlauf

Aufgrund des Schweregrades der Psoriasis vulgaris und der Nagelpsoriasis leiteten wir die Therapie mit Secukinumab 2‑mal 150 mg nach dem regulären Schema mit wöchentlicher Gabe an den Wochen 0, 1, 2, 3 und 4 und anschließender Fortführung in monatlicher Dosis ein.

Innerhalb weniger Wochen wurde eine vollständige Abheilung der Hautveränderungen erreicht (DLQI/PASI = 0), wobei die ausgeprägten Nagelveränderungen bestehen blieben.

**Figure 1:**
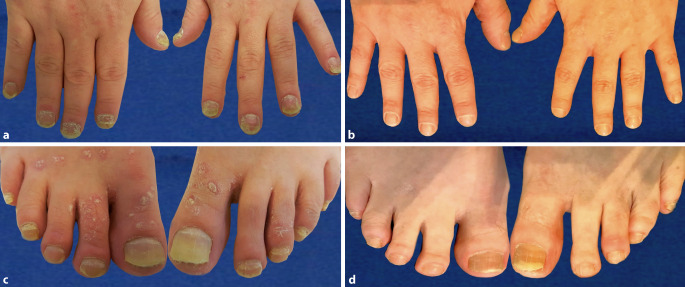

**Figure 2:**
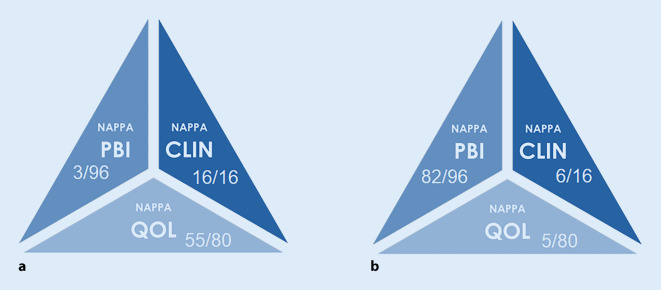

Zeitverzögert, ab der 22. Therapiewoche, kam es zu einer zufriedenstellenden Verbesserung des Nagelbefundes (Abb. 1) einschließlich des NAPPA-Scores mit den Werten: NAPPA-PBI 82/96, NAPPA-QOL 5/80 und NAPPA-CLIN 06/16 (Abb. 2).

## Definition und Hintergrund

Die Nagelbeteiligung ist ein häufiges Symptom der Psoriasis. Etwa 80–90 % der Psoriasis-vulgaris-Patienten klagen über Nagelpsoriasis. Bei Patienten mit Psoriasisarthritis liegt die Inzidenz sogar bei > 60 % [7]. Psychologisch kann die Nagelpsoriasis neben den erheblichen körperlichen Beeinträchtigungen sehr belastend sein. Darüber hinaus beeinträchtigt sie die gesundheitsbezogene Lebensqualität (QoL) der Patienten [9].

Vor diesem Hintergrund ist es umso wichtiger, ein validiertes Messinstrument zur Hand zu haben, das hilft, den Schweregrad der Nagelpsoriasis selbst adäquat zu beurteilen und auch patientenrelevante Aspekte stärker zu berücksichtigen. Derzeit ist keines der gängigen Scoringsysteme für Nagelpsoriasis vollständig validiert [5, 10].

Die Behandlung der Nagelpsoriasis ist häufig eine Herausforderung. Topische und intraläsionale Behandlungen werden als zeitaufwendig empfunden und weisen eine mäßige Wirksamkeit auf. Die aktuelle Evidenz zeigt, dass alle Tumornekrosefaktor-α(TNF-α)-, Interleukin-17- und Interleukin-12/23-Antikörper eine sehr effiziente Therapie für Nagelpsoriasis darstellen [8, 11]. Außerdem wurden die Biologika bei der Erreichung einer vollständigen Heilung der Nagelpsoriasis in Woche 24–26 verglichen. Die Ergebnisse zeigen, dass Ixekizumab die höchste Wahrscheinlichkeit für eine erfolgreiche Nagelpsoriasisbehandlung hat (46,5 %). Die anderen Biologika sind Brodalumab (37,0 %), Adalimumab (28,3 %), Guselkumab (27,7 %), Ustekinumab (20,8 %) und Infliximab (0,8 %) [6].

Als weitere Systemtherapien wurden Methotrexat, Ciclosporin, Acitretin und Apremilast als effiziente Behandlungen für Nagelpsoriasis beobachtet [4, 11].

## Diskussion

Die neuesten Studien haben sich auf die Gültigkeit und Durchführbarkeit von Nagelpsoriasis-Bewertungsinstrumenten konzentriert. Dazu wurden viele verschiedene Tools verwendet. Der NAPSI (Nagelpsoriasis-Schweregrad-Index) wurde beispielsweise als reproduzierbares und objektives Tool beschrieben. Darüber hinaus werden auch das Scoringsystem von Cannavo und der Nagelpsoriasis-Schweregrad-Index von Baran in die klinische Bewertung der psoriatischen Nagelerkrankung einbezogen. Keines der verfügbaren objektiven Maße gibt jedoch Aufschluss über die Belastung des Patienten im täglichen Sozial- und Arbeitsleben oder über die psychische Beeinträchtigung durch Nagelpsoriasis oder Psoriasisarthritis [2].

Daher entwickelte eine internationale Expertengruppe den NAPPA-Score, der zur ersten umfassenden Methodik zur Messung der Patientenbelastung, Patientenbedürfnisse und patientenrelevanten Ergebnisse dient [1].

Der NAPPA-Score besteht aus 3 Komponenten, dem NAPPA-QOL, dem NAPPA-PBI und dem NAPPA-CLIN. Mit dem NAPPA-QOL wird die krankheitsspezifische Lebensqualität des Patienten bewertet (Score von 0 bis 4), und je höher der Score ist, desto bedeutender ist die Auswirkung der Krankheit auf das Leben des Patienten. Der NAPPA-PBI zeigt die Patientenbewertung des therapeutischen Nutzens, die über einen Fragebogen mit 24 Punkten ermittelt wird. Die dritte Komponente, der NAPPA-CLIN, ist eine Kurzform des NAPSI-Scores zur klinischen Beurteilung des Schweregrades der Nagelpsoriasis, der bei der Bewertung aber nur 4 Stellen von insgesamt 20 umfasst, da deren Ergebnis stark mit dem NAPSI-Gesamtwert korreliert [1].

Anhand unseres Fallberichts demonstrieren wir den generellen Einsatz des NAPPA während der Therapie mit Secukinumab. Die Therapie zeigt eine erhebliche Wirksamkeit und eine Verbesserung der Lebensqualität, müsste aber mit anderen mit dem NAPPA erfassten und kontrollierten Nagelbefunden verglichen werden.

In einer kürzlich herausgegebenen Studie wurde nach Analyse von 2 weiteren Studien gezeigt, dass Secukinumab gegenüber Ustekinumab bei Patienten mit Nagelpsoriasis vorzuziehen ist [3]. In diesem Fall konnten individuelle Nagelbehandlungsziele mit dem NAPPA-Score erfasst und überwacht werden, um die Indikatoren und Ergebnisse der Nagelbehandlung präzise zu messen.

Da es wenig wissenschaftliche Daten über den Verlauf von mit dem NAPPA erfassten Nägeln gibt, ist eine Aussage über den zukünftigen Verlauf in unserem Fall schwer einzuordnen. Andererseits erweist sich das Instrument als gut anwendbar. Für die Konstruktionsvalidität ist jedoch eine Validierung erforderlich [1]. Dies gilt für alle wirksamen systemischen Therapien an den Nägeln, wobei wir die dringende Notwendigkeit sehen, diese Datenlücke und die möglicherweise damit verbundene Versorgungslücke bald zu schließen.

## Fazit für die Praxis

Biologika wie Secukinumab haben sich als wirksame und sichere Therapieoptionen bei der Behandlung schwerer Nagelpsoriasis erwiesen. Der NAPPA als dreidimensionales Messinstrument ist bisher nicht ausreichend in solche Studien einbezogen worden. Der NAPPA-Score sollte als Screeningparameter sowohl im dermatologischen Alltag als auch in klinischen Studien mehr Beachtung finden und für ein umfassendes Therapieansprechen immer mitberücksichtigt werden.
